# Artificial intelligence-driven clustering for phenotyping life-threatening prehospital trauma

**DOI:** 10.1186/s13049-026-01553-0

**Published:** 2026-01-15

**Authors:** Rubén Pérez-García, Erik Alonso, Raúl López-Izquierdo, Carlos del Pozo Vegas, Mikel Idoyaga, Asier Losada, José Luis Martín-Conty, Begoña Polonio-López, Ancor Sanz-García, Francisco Martín-Rodríguez

**Affiliations:** 1 Emergency Department, Hospital Universitario Rio Hortega, Valladolid, Spain; 2 Department of Applied Mathematics, University of the Basque Country (UPV/EHU), Bilbao, Spain; 3 Biobizkaia, Bizkaia Health Research Institute, Barakaldo, Spain; 4 Faculty of Medicine, University of Valladolid, Valladolid, Spain; 5 CIBER of Respiratory Diseases, Instituto de Salud Carlos III, Madrid, Spain; 6 Emergency Department, Hospital Clínico Universitario, Valladolid, Spain; 7 Emergency Medical System (Emergentziak-Osakidetza), Basque Health Service, Bilbao, Spain; 8 Faculty of Health Sciences, University of Castilla – La Mancha (UCLM), Talavera de La Reina, Spain; 9 Technological Innovation Applied to Health Research Group (ITAS Group), Faculty of Health Sciences, University of Castilla - La Mancha (UCLM), Talavera de La Reina, Spain; 10 Evaluación de Cuidados de Salud (ECUSAL), Instituto de Investigación Sanitaria de Castilla - La Mancha (IDISCAM), Toledo, Spain; 11 Prehospital Critical Care, Emergency Medical Services (SACYL), Valladolid, Spain

**Keywords:** Prehospital trauma, Blood test biomarkers, Phenotyping, Clustering, Artificial intelligence

## Abstract

**Background:**

Traumatic patients usually suffer from several complex conditions that hinder their risk characterization. The aim of this study was to derive phenotypes of prehospital acute life-threatening trauma via nonsupervised artificial intelligence (AI) clustering methods.

**Methods:**

This was a prospective multicenter study in adult trauma patients treated in prehospital care and transferred to the emergency department. The study included 147 ambulances, 4 helicopters, and 11 hospitals in Spain between 1 January 2021 and 31 August 2024. Epidemiological variables, trauma-related data, baseline vital signs and blood tests were collected. The primary outcome was all-cause 2-day in-hospital mortality.

**Results:**

A total of 1474 patients were included, with a 2-day in-hospital mortality rate of 8.3%. The selected clustering method identified three clusters: the T-1 phenotype comprised 6.9% (101 cases) with a mortality rate of 93.1%, the T-2 phenotype represented 23.6% (348 cases) with a mortality rate of 68.1%, and T-3 represented 69.5% (1,025 cases) with a mortality rate of 10.6%. The T-1 phenotype mainly involves traumatic brain injuries, followed by thoracic trauma and burns; the T-2 phenotype presents a similar distribution; and the T-3 phenotype predominantly involves orthopedic trauma.

**Conclusion:**

The AI method identified three clusters with implications for therapy and outcomes. This novel approach could help emergency medical services characterize trauma patients by providing benefits, treatment and resource optimization.

**Supplementary Information:**

The online version contains supplementary material available at 10.1186/s13049-026-01553-0.

## Key Points

**Question:**

Do prehospital phenotypes characterize trauma patients’ risk of worsening and mortality?

**Findings:**

Unsupervised artificial intelligence methods revealed three phenotypes. Cluster #1 presented a mortality rate of 93.1%, which was associated mainly with traumatic brain injuries, followed by thoracic trauma and burns. Cluster #2 had a 68.1% mortality rate with a similar distribution of traumatic causes. Cluster #3 presented a mortality rate of 10.6%, involving orthopedic traumas.

**Meaning:**

Our results provide specific phenotypes, which could help to improve patients’ care and optimize resources.

**Supplementary Information:**

The online version contains supplementary material available at 10.1186/s13049-026-01553-0.

## Introduction

Quick and timely trauma patient characterization constitutes a major challenge for health care systems, especially for on-scene emergency medical services (EMSs) [1]. Appropriate critical trauma case mapping and subsequent risk stratification for clinical impairment could facilitate complex bedside decision-making processes and guide the adoption of appropriate approaches, e.g., advanced life support maneuvers and referral hospital centers [2]. EMS providers base workflows on objective and structured clinical assessments, limited basal vital signs and complementary tests (biomarkers) [3, 4], and essential basic and advanced life support operations, according to accepted clinical guidelines [5].

Typically, the way to classify high-risk trauma patients has been supported by scores based on physiological criteria, e.g., revised trauma scores [6]; anatomical criteria, e.g., amputations, chest wall instability or deformity, open or depressed skull fracture, paralysis, and pelvic fractures; or even injury mechanisms, e.g., ejection from the vehicle, extrication > 20 min, death of an occupant in the same vehicle, impact speed > 80 km/h, pedestrian struck, and falls > 3 m [7, 8].

In addition, major trauma during the ultra-acute stage typically involves sudden hemodynamic changes; therefore, additional prehospital care tools to properly categorize acute life-threatening trauma can make a significant difference [9, 10]. In this sense, in in-hospital care, phenotyping is commonly performed for the assessment and treatment of chronic diseases, e.g., chronic obstructive pulmonary disease, heart failure or even acute life-threatening diseases such as sepsis or severe acute respiratory syndrome coronavirus 2 (SARS-CoV-2) [11].

The emergence of artificial intelligence (AI) techniques has allowed clustering methods based on clinical criteria to be redefined and, sometimes, enhanced [12, 13]. Trauma is a challenging and heterogeneous condition, and identifying phenotypes involving acute life-threatening trauma, with different pathophysiological routes and determinants, from the outset would help to provide a tailor-made focus, optimizing interventions and ultimately yielding superior outcomes.

The primary purpose of this study was to derive phenotypes associated with prehospital acute life-threatening trauma via nonsupervised AI methods. The secondary purpose was to identify lethal clinical phenotypes with relatively high short-term clinical deterioration rates.

## Methods

### Study design and settings

This was a prospective, multicenter, ambulance-based, ongoing study in trauma patients treated in prehospital care and transferred to the emergency department (ED).

Patients were consecutively and prospectively recruited from three studies conducted under comparable protocols: “Advanced Precision Scoring-System for Prehospital Critical Care Based on Artificial Intelligence (APPS) study”, “Identification of biomarkers of clinical-risk deterioration in prehospital care—preBIO study", and “PREdiction of the risk of early clinical deterioration in prehospital emergency patients through artificial intelligence techniques—predecIA study”. The studies were granted the approval of the institutional review board of the Public Health Service (reference: 23-PI027, PI217-20, and EAG-PRE-2024–01). This study follows the STrengthening the Reporting of OBservational studies in Epidemiology (STROBE) guidelines (Supplementary data p3).

The study comprised 15 advanced life support (ALS) units, 4 helicopters of emergency medical service (HEMS), 132 basic life support (BLS) units, 2 emergency dispatch centers, and 11 hospitals from two different EMS systems (SACYL and Osakidetza, operated by the National Health System, all in Spain), between ^1^ January 2021 and ^31^ August 2024, providing around-the-clock service to a population of 3,041,937 inhabitants in cities, suburbs, and rural areas in 8 different regions.

BLSs are typically operated by two emergency medical technicians (EMTs), and ALSs and HEMSs are additionally staffed with an emergency registered nurse (ERN) and a physician (for more information concerning the EMS system, see supplementary data on p5).

Informed consent was obtained by the ERN on a routine basis during the first encounter with the patient or legal guardian. When the patient, either due to his critical condition or suboptimal awareness, was unable to comprehend or sign the document, informed consent was obtained by an associate researcher on second request in the EDs.

### Population

Adults (≥ 18 years old) with a trauma background (blunt and/or penetrating) evaluated on a mandatory basis by the ALS or HEMS physician and subsequently referred to the hospital via the BLS, ALS or HEMS were consecutively included in the study.

Minors, nontrauma patients, pregnant women (evident or suspected), those whose cardiorespiratory arrest did not recover on-scene, who were unavailable for prehospital blood tests (e.g., failure of point-of-care testing, inaccessible venous line blood collection), or who lacked informed consent were excluded.

### Outcomes

The primary outcome was 2-day in-hospital mortality (all-cause). In such a short-term time frame, the cause of fatality should be directly linked to the triggering cause of EMS system care [14]. The principal investigator double-checked all patients for 2-day mortality. In the presence of missing data, the case was excluded from the analysis.

The secondary outcomes included prehospital massive hemorrhage control (limb tourniquets, hemostatic gauze, injectable hemostatic agents, wound closure devices and pelvic compression devices), invasive mechanical ventilation (IMV), needle decompression of the chest, crystalloid infusion, tranexamic acid, noradrenaline, analgesics, hypnotics, and neuromuscular blocking agent administration. Finally, the in-hospital outcomes included IMV, tranexamic acid and noradrenaline administration, blood transfusion, emergent surgery, yes/no intensive care unit (ICU) admission, and 30-day in-hospital mortality.

### Predictors and data abstraction

Epidemiological variables (sex and age), trauma-related data (mechanism, type of trauma, isochrones, etc.), baseline vital signs (respiratory rate, oxygen saturation, fraction of inspired oxygen, blood pressure, heart rate, temperature and Glasgow Coma Scale (GCS)) and blood test data (pH, partial pressure of carbon dioxide, bicarbonate, base excess, sodium, potassium, calcium, chlorine, hemoglobin, glucose, lactate, creatinine and urea) were collected and logged by the ERN during the first EMS encounter with the patient and prior to performing any intervention on the victim. The LifePAK® 15 monitor-defibrillator (Physio-Control, Inc., Redmond, USA) and CORPULS3T (GS Elektromedizinische Geräte G. Stemple GmbH, Germany) were employed to measure vital signs, and the Epoc® Blood Analysis System (Siemens Healthcare GmbH, Erlangen Germany) was used for prehospital blood analyses. The physician entered the EMS registry with the need for hemorrhage control, mechanical ventilation, and drug administration.

After a 30-day follow-up period postindex event (prehospital care), an associate investigator from every hospital, by reviewing electronic medical records, collected hospital follow-up data, including mechanical ventilation, tranexamic or noradrenaline administration, blood transfusion, emergency surgery, intensive care unit (ICU) admission, and 2- and 30-day in-hospital mortality, 17 required comorbidities were collected to determine the age-adjusted Charlson comorbidity index (ACCI), and the injury severity score (ISS) was subsequently estimated (Supplementary Table S1). Next, all the scores tested were calculated via the prospectively collected data. Neither EMS providers nor hospital research associates were aware of the scores under consideration.

### Statistical analysis

Descriptive and bivariate statistics for the outcome variables were assessed by the t test, the Mann‒Whitney U test or the chi‒square test, whenever appropriate. Absolute values and percentages were used for categorical variables, and medians and interquartile ranges (IQRs) were used for continuous variables that were not normally distributed. Data collection, missing value management and sample size calculations based on recent studies [15] are reported in supplementary data p9.

Clustering was performed via four different methods based on unsupervised machine learning methods (note that for all the methods, the number of clusters was fixed to three on the basis of clinical criteria). Details regarding the methodology can be found in supplementary data p9.

Agreement between clustering methods was compared by including patients classified in the three clusters in a 3 × 3 contingency table and assessed by a chi-square test, by Cohen’s kappa (for the kappa interpretation, a kappa > 0.8 could be considered perfect agreement), and by the adjusted Rand index (which is interpreted in a similar way to Cohen’s kappa, i.e., values closest to 1 indicate greater agreement). Only the method with the highest agreement vs the other methods was selected for further analysis.

The differences between clusters were explored by univariate analysis, which was further evaluated for early warning scores by graphically representing the values of each score for each cluster and by a post hoc pairwise comparison.

All calculations and analyses were performed by our own codes in R, version 4.2.2 (http://www.R-project.org; the R Foundation for Statistical Computing, Vienna, Austria), Python, version 3.12.7 (https://www.python.org/), and MATLAB, version R2022b (Natick, MA, USA).

## Results

**Fig. 1:**
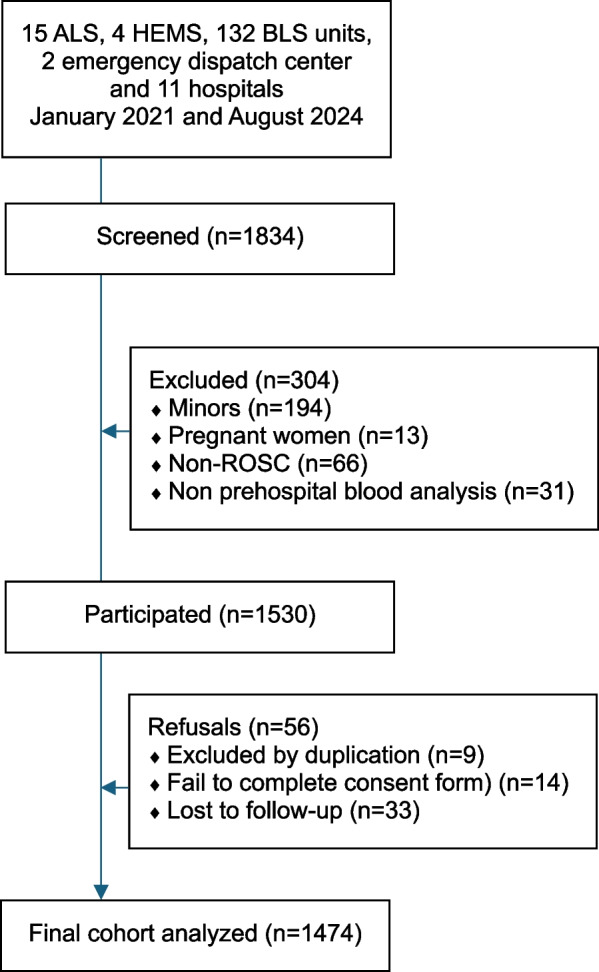
Study flowchart. *Abbreviations*: ALS: advanced life support unit; HEMS: helicopter emergency medical service; BLS: basic life support unit; ROSC: recovery of spontaneous circulation

A total of 1474 trauma patients were ultimately analyzed (see Fig. 1). The median age was 53 years (IQR: 37–70), 34.4% (507 patients) were female, and the 2-day in-hospital mortality rate was 8.3% (123 patients).

Agreement between methods was slight/fairly good, as shown by the results of the adjusted Rand index, Cohen's kappa and chi-square (Table 1). Therefore, based on the results, clustering method 2 (Beta) presented all the results. Briefly, clustering method Beta used the K-means algorithm, a straightforward and robust method for partitioning data into K distinct, non-overlapping groups. All available variables were included in the analysis, and the number of clusters (K = 3) was determined based on clinical criteria. Following clustering, principal component analysis (PCA) was conducted to explore the relative contribution of each variable to the final cluster assignment.

**Table 1:** Association between the clustering methods tested

Clustering method	Clustering method	Contingency tables				Adjusted Rand Index	Cohen’s kappa		Chi square statistic
Alfa	Beta		1	2	3	0.27	0.38		792.9
		1	85	71	14				
		2	14	191	297				
		3	2	86	714				
Alfa	Gamma		1	2	3	0.26		0.40	657.4
		1	96	74	0				
		2	42	239	221				
		3	25	133	644				
Afa	Delta		1	2	3	0.29	0.38		813.9
		1	81	79	10				
		2	13	190	299				
		3	1	76	725				
Beta	Gamma		1	2	3	0.54	0.64		1311.1
		1	89	12	0				
		2	58	272	18				
		3	16	162	847				
Beta	Delta		1	2	3	0.80	0.86		2388.7
		1	94	7	0				
		2	1	303	44				
		3	0	35	990				
Gamma	Delta		1	2	3	0.48	0.60		1205.0
		1	86	55	22				
		2	9	258	179				
		3	0	32	833				

Phenotyping based on the Beta AI criteria revealed three well-defined clinical clusters (*T*−1, *T*−2 and *T*−3).

**Fig. 2:**
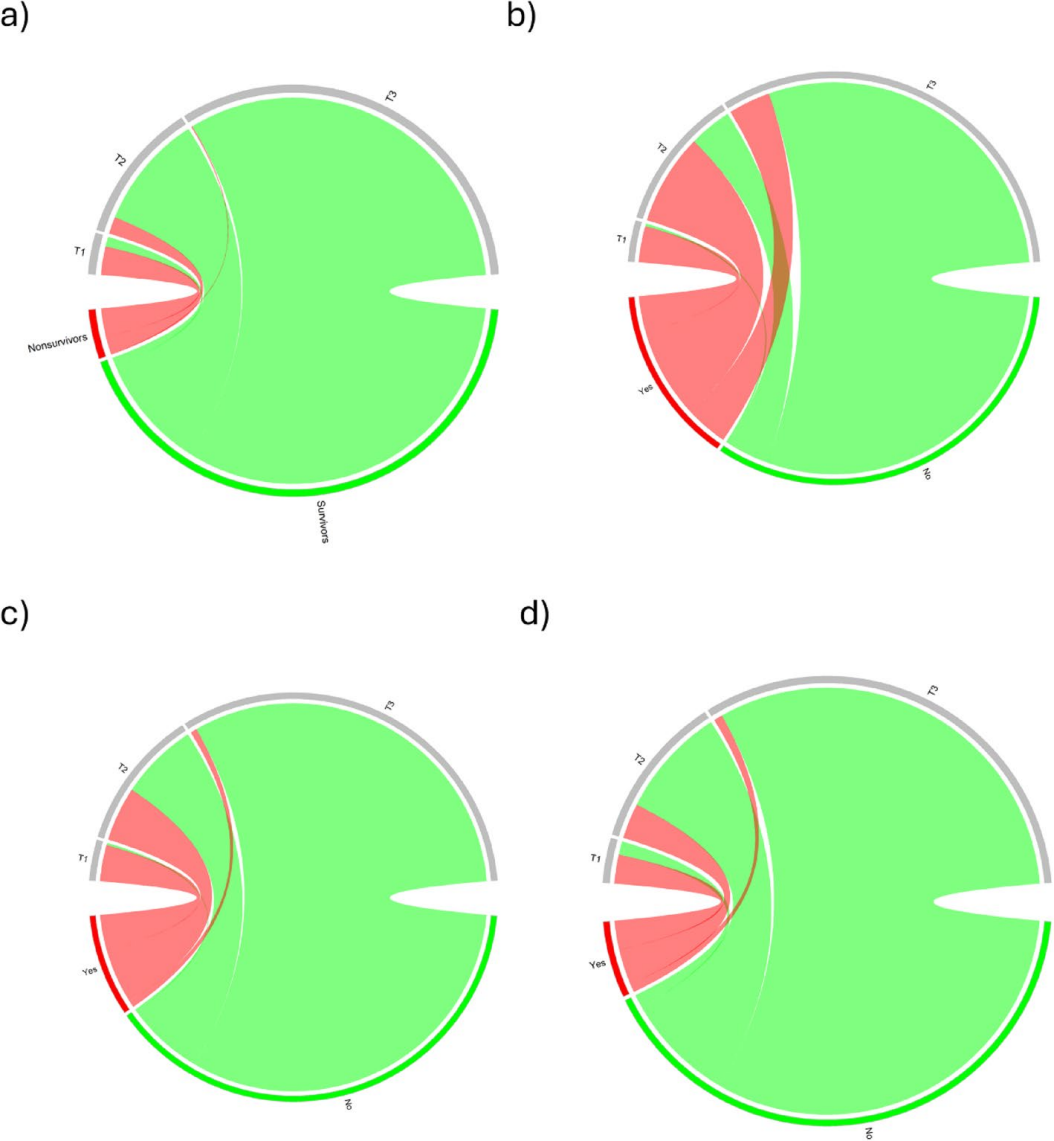
Chord diagram for the generation of clusters for each outcome. **a** 2-day mortality, **b** ICU admission, **c** prehospital mechanical ventilation, and **d** blood transfusion

The distribution of outcomes per cluster is shown in Fig. 2. The *T*−1 phenotype was present in 6.9% (101 patients), with a median age of 56 years (IQR: 40–74), and 34.7% (35 patients) of the patients were females. Vital signs indicate severe desaturation, a propensity for hemodynamic instability and a decreased consciousness level. Prehospital blood tests indicated a pronounced tendency toward acidosis, with a median pH of 6.99 (IQR: 6.88–7.12), decreased base excess and elevated lactate (9.23 mmol/L; IQR 6.87–13.1). Similarly, the metabolic-oxidative stress response to trauma, with signs of hyperglycemia and kidney overload, is highlighted in this cluster (Table 2). The *T*−1 phenotype reported the most life-saving interventions, with 94.1% prehospital mechanical ventilation, 63.4% tranexamic administration or a median of 1000 ml (IQR: 120–1250) of infused crystalloids. The in-hospital trend was confirmed, with mechanical ventilation rates of 95%, noradrenaline administration rates of 87.1%, blood transfusion rates of 68.3%, emergency surgery rates of 58.4% and, as expected, ICU-admission and 2- and 30-day in-hospital mortality rates of 93.1%, 74.3% and 82.2%, respectively.

**Table 2:** Baseline clinical characteristics and outcomes of the study population

		Artificial intelligence phenotypes			
	Total	*T*−1	*T*−2	*T*−3	*p* value^b^
No. (%) with data^a^	1474 (100)	101 (6.9)	348 (23.6)	1025 (69.5)	N.A
Sex, female	507 (34.4)	35 (34.7)	107 (30.7)	365 (35.6)	0.139
Age	53 (37–70)	56 (40–74)	59 (45–78)	50 (36–65)	< 0.001
Basal vital signs					
RR, breaths/min	18 (15–22)	18 (7–28)	19 (16–25)	18 (15–20)	< 0.001
SpO2, %	97 (94–99)	81 (66–90)	95 (91–97)	98 (96–99)	< 0.001
FiO2, %	0.21 (0.21–0.21)	0.21 (0.21–0.5)	0.21 (0.21–0.21)	0.21 (0.21–0.21)	< 0.001
SBP, mmHg	132 (116–147)	88 (70–113)	130 (107–150)	135 (120–148)	< 0.001
DBP, mmHg	79 (67–90)	54 (38–61)	76 (61–88)	80 (70–90)	< 0.001
Heart rate, beats/min	85 (72–101)	115 (89–133)	91 (76–113)	81 (75–91)	< 0.001
Temperature, °C	36 (35.7–36.3)	35.7 (34.7–36.1)	36 (35.5–36.1)	36 (35.9–36.4)	< 0.001
Ocular GCS, points	4 (4–4)	1 (1–2)	3 (2–4)	4 (4–4)	< 0.001
Verbal GCS, points	5 (5–5)	1 (1–2)	4 (1–5)	5 (5–5)	< 0.001
Motor GCS, points	6 (6–6)	3 (1–4)	6 (4–6)	6 (6–6)	< 0.001
Prehospital blood test					
pH	7.37 (7.32–7.42)	6.99 (6.88–7.12)	7.31 (7.22–7.36)	7.39 (7.36–7.43)	< 0.001
pCO2, mmHg	38 (32–44)	45 (34–66)	42 (34–48)	36 (32–42)	< 0.001
Bicarbonate, mEq	23.1 (20.6–25.7)	15.4 (13.2–17.9)	20.4 (18.4–22.4)	24.1 (22.3–26.5)	< 0.001
BE, mmol/L	−0.5 (−3; 1.2)	−10.7 (−15; −7.5)	−3.2 (−5.8; −1)	0.9 (−1.1; 1.9)	< 0.001
Sodium, mmol/L	139 (137–141)	140 (135–141)	140 (137–142)	139 (137–140)	0.129
Potassium, mmol/L	4.1 (3.8–4.4)	4.7 (3.9–5.7)	4 (3.7–4.4)	4.1 (3.8–4.4)	< 0.001
Calcium, mmol/L	1.13 (1.05–1.21)	1.01 (0.88–1.19)	1.11 (1.01–1.11)	1.15 (1.09–1.21)	< 0.001
Chlorine, mmol/L	103 (100–106)	105 (102–109)	105 (102–108)	102 (100–105)	< 0.001
Hemoglobin, g/dL	13.8 (12.6–15.5)	9.5 (7.6–12.4)	13.2 (11.8–14.8)	14.2 (13.1–15.7)	< 0.001
Glucose, mg/dL	121 (103–150)	193 (132–243)	143 (115–187)	114 (100–134)	< 0.001
Lactate, mmol/L	2.64 (1.77–3.71)	9.23 (6.87–13.1)	3.81 (2.97–5.44)	2.13 (1.37–2.97)	< 0.001
Creatinine, mg/dL	0.87 (0.75–1.09)	1.65 (1.14–2.18)	0.99 (0.85–1.27)	0.81 (0.71–0.94)	< 0.001
Urea, mg/dL	31.2 (23.4–44.4)	63.6 (50.7–108.1)	40.8 (28.3–56.4)	27.3 (22.2–37.2)	< 0.001
Prehospital outcomes					
Hemorrhage control^c^	159 (10.8)	35 (34.7)	57 (16.4)	67 (6.5)	< 0.001
Mechanical ventilation	255 (17.3)	95 (94.1)	143 (41.1)	17 (1.7)	< 0.001
Tranexamic	181 (12.3)	64 (63.4)	87 (25)	30 (2.9)	< 0.001
Noradrenaline	86 (5.8)	60 (59.4)	24 (6.9)	2 (0.2)	< 0.001
Crystalloids, ml	250 (250–250)	1000 (120–1250)	250 (250–500)	250 (250–250)	< 0.001
NDC	80 (5.4)	29 (28.7)	38 (10.9)	13 (1.3)	< 0.001
Minor analgesic^d^	637 (43.2)	10 (9.9)	122 (35.1)	505 (49.3)	< 0.001
Major analgesic^e^	785 (53.3)	83 (82.2)	263 (75.6)	439 (42.8)	< 0.001
Hypnotic^f^	379 (25.7)	93 (92.1)	192 (55.2)	93 (9.1)	< 0.001
NBA^g^	251 (17)	93 (92.1)	141 (40.5)	17 (1.7)	< 0.001
Hospital outcomes					
Mechanical ventilation	327 (22.2)	96 (95)	189 (54.3)	42 (4.1)	< 0.001
Tranexamic	72 (4.9)	38 (37.6)	29 (8.3)	5 (0.5)	< 0.001
Noradrenaline	186 (12.7)	88 (87.1)	86 (24.7)	12 (1.2)	< 0.001
Blood transfusion	174 (11.8)	69 (68.3)	84 (24.1)	21 (2)	< 0.001
Emergent surgery	454 (30.8)	59 (58.4)	184 (52.9)	211 (20.6)	< 0.001
aCCI, points	1 (0–4)	3 (0–6)	2 (0–5)	1 (0–3)	< 0.001
ISS, points	9 (4–16)	41 (25–51)	16 (9–25)	5 (4–12)	< 0.001
ICU-admission	440 (29.9)	94 (93.1)	237 (68.1)	109 (10.6)	< 0.001
2-day mortality	123 (8.3)	75 (74.3)	45 (12.9)	3 (0.3)	< 0.001
30-day mortality	182 (12.3)	83 (82.2)	87 (25)	12 (1.2)	< 0.001

*Abbreviations*: *NA* not applicable, *RR* respiratory rate, *SPO2* oxygen saturation, *FIO2* fraction of inspired oxygen, *SBP* systolic blood pressure, *DBP* diastolic blood pressure, *GCS* Glasgow Coma Scale, *pCO2* partial pressure of carbon dioxide, *BE* base excess, *NDC* needle decompression of the chest, *NBA* neuromuscular blocking agent, *aCCI* adjusted Charlson comorbidity index, *ISS* injury severity score, *ICU* intensive care unit

^a^Values expressed as total number (percentage) and medians [25th percentile–75th percentile], as appropriate

^b^The Mann‒Whitney U test, t test or chi-squared test was used as appropriate

^c^Hemorrhage control: limb tourniquets, hemostatic gauze, injectable hemostatic agents, wound closure devices and pelvic compression devices

^d^Minor analgesic: aspirin, dexketoprofen, diclofenac, metamizole, and acetaminophen

^e^Major analgesic: morphine, fentanyl, meperidine, tramadol, and ketamine

^f^Hypnotic: diacepam, midazolam, etomidate, and propofol

^g^Neuromuscular blocking agent: suxamethonium, rocuronium, and cisatracurium

The T-*2* phenotype was represented by 23.6% (348 patients), with a median age of 59 years (IQR: 45–78), and 30.7% (107 patients) of the patients were females.

This cluster revealed normalization of both vital signs and disturbed analytical parameters in the *T*−1 phenotype. On-scene life-saving interventions were significantly lower than those in the T-1 phenotype (Table 2), with ICU-admission and in-hospital mortality rates at 2 and 30 days of 68.1%, 12.9% and 25%, respectively.

Finally, the T-3 phenotype accounted for 69.5% (1025 patients), with a median age of 50 years (IQR: 36–65), and 35.6% (365 patients) were females.

This cluster reported no statistically significant abnormalities in the studied variables, a drastic drop in on-scene life-saving interventions and ICU-admission and in-hospital mortality rates at 2 and 30 days of 10.6%, 0.3% and 1.2%, respectively (Table 2).

Among those with the T-1 phenotype, 37.6% (38 patients) experienced traumatic brain injuries, followed by thoracic trauma and burns; among those with the T-2 phenotype, the distribution was similar, but orthopedic trauma was the third most common, and among those with the T-3 phenotype, orthopedic trauma was the most common (Supplementary Figure S1). With respect to mortality and type of trauma, T-1 patients presented higher mortality rates for the thorax and abdomen; T-2 mortality rates were mainly associated with traumatic brain injuries; and T-3 mortality rates were only associated with traumatic brain injuries (Supplementary Figure S1). The epidemiological characteristics of the study population can be found in Table 3.

**Table 3:** Epidemiological characteristics of the study population

		Artificial intelligence phenotypes			
	Total	*T*−1	*T*−2	*T*−3	*p* value^b^
No. (%) with data^a^	1474 (100)	101 (6.9)	348 (23.6)	1025 (69.5)	N.A
Sex, female					
Female	507 (34.4)	35 (34.7)	107 (30.7)	365 (35.6)	0.139
Male	967 (65.6)	66 (65.3)	241 (69.3)	660 (64.4)	
Age	53 (37–70)	56 (40–74)	59 (45–78)	50 (36–65)	< 0.001
18–49	637 (43.2)	34 (3.7)	108 (31.1)	495 (48.3)	< 0.001
50–74	543 (36.8)	42 (41.6)	133 (38.2)	368 (35.9)	
> 75	294 (19.9)	25 (24.8)	107 (30.7)	162 (15.8)	
Trauma mechanism					
Road crash	415 (28.2)	28 (27.7)	96 (27.6)	291 (28.4)	0.158
Hit and run	126 (8.5)	12 (11.9)	18 (5.2)	96 (9.4)	
Falls	420 (28.5)	12 (11.9)	116 (33.3)	292 (28.5)	
Precipitate	116 (7.9)	13 (12.9)	41 (11.8)	62 (6)	
Sports accident	119 (8.1)	0 (0)	17 (4.9)	102 (10)	
Wounds and injuries	191 (13)	16 (15.8)	33 (9.5)	142 (13.9)	
Suicide attempt	87 (5.9)	20 (19.8)	27 (7.8)	40 (3.9)	
Zone					
Urban	952 (64.6)	55 (54.5)	190 (54.6)	707 (69)	< 0.001
Rural	522 (35.4)	46 (45.5)	158 (45.4)	318 (31)	
Arrival time, min	11 (8–17)	12 (8–25)	12 (8–22)	10 (8–15)	< 0.001
< 10 min	692 (46.9)	37 (36.6)	134 (38.5)	521 (50.8)	< 0.001
11–30 min	675 (45.8)	50 (49.9)	170 (48.9)	455 (44.4)	
> 30 min	107 (7.3)	14 (13.9)	44 (12.6)	49 (4.8)	
Support time, min	30 (23–40)	43 (32–50)	33 (25–44)	28 (21–36)	< 0.001
< 10 min	21 (1.4)	0 (0)	6 (1.7)	15 (1.5)	< 0.001
11–30 min	736 (49.9)	20 (19.8)	142 (4.8)	574 (56)	
> 30 min	717 (48.6)	81 (80.2)	200 (57.5)	436 (42.5)	
Evacuation time, min	13 (9–21)	17 (11–30)	16 (10–26)	12 (8–19)	< 0.001
< 10 min	532 (36.1)	24 (23.8)	100 (28.7)	408 (39.8)	< 0.001
11–30 min	781 (53)	52 (51.5)	181 (52)	548 (53.5)	
> 30 min	161 (10.9)	25 (24.8)	67 (19.3)	69 (6.7)	
Total time, hour and min	0:57 (0:44–1:15)	1:16 (0:58–1:42)	1:04 (0:48–1:32)	0:54 (0:43–1:07)	< 0.001
Evacuation carrier					
Advanced life support	969 (65.7)	83 (82.2)	280 (80.5)	606 (59.1)	< 0.001
HEMS	55 (3.7)	17 (6.8)	31 (8.9)	7 (0.7)	
Basic life support	450 (30.5)	1 (1)	37 (10.6)	412 (40.2)	
Trauma-related events					
Primary care on-scene	283 (19.2)	33 (32.7)	99 (28.4)	151 (14.7)	< 0.001
Nursing homes	90 (6.1)	5 (0.5)	35 (10.1)	50 (5.9)	0.003
Rescue	275 (18.7)	35 (34.7)	82 (23.6)	158 (15.4)	< 0.001
Intoxication	102 (6.9)	7 (6.9)	30 (8.6)	65 (6.3)	0.186
CA with ROSC	23 (1.6)	20 (19.8)	3 (0.9)	0 (0)	< 0.001
Primary trauma					
Trauma brain injury	497 (33.7)	38 (37.6)	170 (48.9)	289 (28.2)	< 0.001
Spinal	114 (7.7)	8 (7.9)	11 (3.2)	95 (9.3)	
Head and neck	31 (2.1)	0 (0)	3 (0.9)	28 (2.7)	
Thorax	206 (14)	21 (20.8)	58 (16.7)	127 (12.4)	
Abdomen	68 (4.6)	13 (12.9)	26 (7.5)	29 (2.8)	
Pelvic	60 (4.1)	8 (7.9)	23 (6.6)	29 (2.8)	
Orthopedic	350 (23.7)	2 (2)	33 (9.5)	315 (30.7)	
Burns	60 (4.1)	11 (10.9)	19 (5.5)	30 (2.9)	
Polytraumatized	88 (6)	83 (82.1)	5 (1.4)	0 (0)	

*Abbreviations*: *NA* not applicable, *HEMS* helicopter emergency medical service, *CA* cardiac arrest, *ROSC* return of spontaneous circulation

^a^Values expressed as total number (fraction) and medians [25th percentile–75th percentile], as appropriate

^b^The Mann‒Whitney U test, t test or chi‒square test was used as appropriate

The clustering procedure was followed by a PCA aiming at analyzing the individual relative importance of each predictor in the cluster assignment. Supplementary Figure S2 represents the percentage of variance explained by each of the 31 principal components in the data. Supplementary Figure S3 shows a biplot of the first two principal components representing both the principal component scores and the loading vectors. Similarly, Supplementary Figure S4 depicts a heatmap of the loading vectors of all the principal components. The first principal component explained approximately 27% of the variance inherent in the data, which accounted for at least three times more variance than the remaining principal components did. Nevertheless, its loading vector revealed no predominant variable, placing approximately equal weights on pH, lactate, bicarbonate, the two base excess and the three GCS variables, whereas substantially less weight was assigned to the remaining variables. Conversely, the second principal component, containing approximately 8% of the variance in the data, placed greater weights on total carbon dioxide content, blood urea nitrogen, urea, age, and creatinine.

## Discussion

To the best of our knowledge, this is the first prehospital study to characterize different phenotypes in acute life-threatening trauma patients.

On the basis of the analysis of 25 epidemiological, clinical and biomarker variables collected by EMS providers on scene or *en route* and disregarding the outcome, three well-defined clinical clusters have been established. The *T*−1 phenotype was associated with an increased risk of short-term mortality, ICU admission and life-saving interventions (pre- and in-hospital). Lethality in the *T*−1 phenotype was six times greater than that observed in the *T*−2 phenotype (2-day mortality: 74.3% vs 12.9%) and substantially higher than in the T-3 phenotype (2-day mortality: 74.3% vs 0.3%).

Recently, clinical characterization-related research has appeared regularly. This approach is revolutionizing clinical medicine, allowing detailed profiling of health conditions, earlier and precise diagnosis, targeted therapies or healthcare process improvement [16]. Focusing specifically on trauma patients, several studies are used to identify severe cases, e.g., Tachino et al. [17] retrospectively studied the association between tranexamic acid therapy and in-hospital mortality in 8 clinical phenotypes involving blunt trauma patients. Reilly et al. [18] identified 3 different phenotypes of acute respiratory distress syndrome after major trauma on the basis of plasma biomarkers monitored in the ICU. Alternatively, Johansson et al. [19], via SHINE biomarker (syndecan-1, soluble thrombomodulin, adrenaline) screening, established one phenotype with a 22% prevalence of severe hypocoagulability and hyperfibrinolysis and, consequently, associated fatality. The aforementioned studies were carried out in hospital centers; as a result, the extrapolation of outcomes to prehospital critical care is challenging. In this context, phenotyping is emerging for the classification and rapid identification of prehospital critical patients [20, 21]. However, no study based on prehospital biomarkers in the hyperacute stage has classified acute life-threatening trauma.

Our study revealed three phenotypes with well-defined clinical and prognostic characteristics. The *T-1* phenotype accounted for 7% of the total cases (51.5% −52 cases- were polytrauma vs. 48.5% −49 cases- which involved polytrauma with associated traumatic brain injury); however, the short-term mortality rate was high (74%), clearly surpassing those of the T-2 and T-3 phenotypes (13% and 0.3%, respectively). Hemodynamic instability, pronounced lactic acidosis and renal failure may contribute to the lethality gap, leading to rapid worsening and the onset of shock with multiorgan failure syndrome [22, 23]. The predominant damage caused by the T-1 and T-2 phenotypes was traumatic brain injury; additionally, the *T-1* phenotype was associated with a significant percentage of injuries (thorax, abdomen and burns), and multiple types of trauma were associated with the injury severity score (ISS), with peak values also observed for the *T-1* phenotype [24]. This higher mortality in the most lethal cluster may also be related to the prevalence of chronic medical conditions. Indeed, despite the *T-2* phenotype having an older median age, the *T-1* phenotype scored a higher ACCi, which is consistent with the findings of similar studies that show that the burden comorbidity is more decisive than age per se [25].

As expected, the *T-2* phenotype exhibited vital and analytical indicators with slight disturbances, showing an evident tendency toward normalization compared with the *T-1* phenotype but with worse performance than the *T-3* phenotype. The data for life-saving interventions (pre- and in-hospital), ICU admission and short-term mortality in this cluster, although not negligible (2-day mortality rate of 12.9%), compared with the *T-1* phenotype, were quite inferior, as were the scores on the ISS and ACCi. Falls were one of the dominant causes of damage in this cluster, leading to traumatic brain injury primarily without other associated injuries (representing the leading cause of mortality in this cohort), explaining the lower mortality rate than that associated with the *T-1* phenotype [26]. Finally, the *T-3* phenotype yielded the best overall outcomes (2-day mortality rate of 0.3%), paradoxically being the largest cluster.

The clinical use of phenotyping is a valuable resource for providing personalized healthcare based on unique patient profiles. In trauma patients, rapid on-scene or *en-route* categorization helps to detect low-survival cases [27]. Proper labeling may help guide follow-up strategies for polytraumatized, complex and fast-changing patients, especially when patients are deciding on the most suitable hospital with appropriate facilities, a crucial step often based on poor clinical data [28]. Similarly, phenotyping by EMS providers provides guidance on life-saving interventions, the appropriate treatment response and even the potential for systemic inflammatory response syndrome from minute zero [29].

Early warning scores and biomarkers are key to understanding the individual risk associated with every patient and recognizing potential clinical impairment. In addition, defining clinical clusters will enable the design of strategies to provide individual support. Although the physio-pathological processes operating in the different phenotypes are not fully understood, evidence suggests that different outcomes are likely to result depending on the phenotype. In other words, based on prehospital critical care parameters, EMS providers could establish prehospital phenotypes, ascertain the respective lethality, and identify promptly patients at risk of acute life-threatening trauma, thereby supporting early decision-making and potential life-saving interventions [20, 30].

AI-driven phenotyping offers a complementary approach to traditional risk stratification methods, which often rely on a limited set of physiological or anatomical indicators. By integrating multiple data streams (such as vital signs, blood gas measures, and metabolic markers) phenotype-based classification may provide a broader view of patient status. While this approach is promising, further research is required to determine its clinical utility in the prehospital management of life-threatening trauma.

This approach enables EMS providers to move beyond reactive care toward predictive and personalized interventions. By identifying distinct pathophysiological phenotypes associated with varying mortality risks, clinicians can tailor treatment intensity, anticipate potential complications, and make informed decisions regarding transport destinations and hospital resource activation. Phenotyping may help organize raw clinical data into more interpretable patterns that could support early recognition of patients at higher risk of severe outcomes. Although its direct impact on clinical decision-making remains to be established, these patterns may eventually assist in identifying patients who might benefit from advanced interventions or referral to specialized centers.

Integrating AI-derived phenotyping into prehospital decision support tools is an area of growing interest. As point-of-care technologies and computational methods continue to evolve, real-time phenotype assessment may become feasible in the future. However, prospective validation studies are necessary to determine whether phenotype-guided strategies meaningfully improve clinical outcomes or resource utilization, and whether they can be translated into evidence-based protocols for prehospital trauma management.

This study has several limitations. First, although a convenience sample was used, case recruitment was conducted continuously (24/7/365) across multiple EMS systems and geographic settings to reduce selection bias. Nonetheless, the use of a non-probability sample may limit the generalizability of the findings to other prehospital systems with different organizational structures, triage protocols, or resource availability. Second, data extractors were unblinded, which may introduce information bias. To minimize this risk, EMS providers did not have access to hospital follow-up data and ED providers did not have access to prehospital data; only the principal investigator and data manager handled the complete dataset and phenotype derivation. However, some subjective elements of data recording cannot be fully excluded. Third, although point-of-care testing is increasingly used in prehospital care, the availability of specific biomarkers varies across EMS systems. As a result, replication in settings without similar equipment or training may be challenging, and external validation will be necessary to assess reproducibility and applicability. Fourth, analytical variables collected in the hyperacute on-scene phase may not fully reflect the evolving clinical condition. Some parameters, such as hemoglobin, may remain unchanged despite active bleeding due to the short time interval from injury to sampling, potentially influencing cluster assignment and phenotype interpretation. Fifth, because this was an observational exploratory phenotyping study, the identified clusters should not be interpreted as causal or definitive. Unsupervised clustering may be sensitive to the choice of variables and modeling assumptions, and therefore the stability of phenotypes requires confirmation in independent cohorts. Finally, part of the study period overlapped with the SARS-CoV-2 pandemic, during which trauma epidemiology changed markedly due to mobility restrictions. The reduced incidence of polytrauma events during this time may affect the representativeness of the sample, and these temporal effects should be considered in future longitudinal or epidemiological analyses.

In summary, trauma patients managed by EMS can be categorized into 3 clinical phenotypes, with implications for therapy and outcomes. The *T*−1 phenotype was associated with worse outcomes, more intensive care unit (ICU) admissions, more life-saving interventions (both prehospital and in-hospital) and short-term mortality. This layering could help personalize medical treatment, and the implementation of precision medicine from the first point of care represents a novel approach to prehospital critical care.

## Supplementary Information

Supplementary Material 1.

## Data Availability

The datasets analyzed during the current study, including the deidentified participant data, are available from the corresponding author on reasonable request.
