# Supplementary material for: Artificial intelligence-driven clustering for phenotyping life-threatening prehospital trauma
Source: Scand J Trauma Resusc Emerg Med. 2026 Jan 15;34:30. doi: 10.1186/s13049-026-01553-0 (PMC12892782; doi:10.1186/s13049-026-01553-0)

**Supplementary data**

Table of contents

Supplementary methods…………………………………………………………………3

Strengthening the Reporting of Observational Studies in Epidemiology (STROBE) Statement………………………...……………………………….…3

Details about the Emergency medical services……...………………………….5

- Supplementary Table S1: Injury severity Score and Age-adjusted Charlson Comorbidity Index calculation………………………………………….……....6

Data collection, sample size calculation and clustering methods….……………9

Supplementary Results…………………………………………………………………10

- Supplemental Figure S1. Chord diagram for mortality and type of trauma…..10

- Supplementary Figure S2: percentage of variance explained by each of the principal components……..…………………………………………………….11

- Supplementary Figure S3: Biplot of the first two principal components representing both the principal component scores and the loading vectors…….12

- Supplementary Figure S4: Heatmap of the loading vectors (φ) of all the principal components…………………………………………………………...13

**Supplementary Methods**

STROBE Statement—checklist of items that should be included in reports of observational studies

|  | Item No | Recommendation | Page  No |
| --- | --- | --- | --- |
| **Title and abstract** | 1 | (*a*) Indicate the study’s design with a commonly used term in the title or the abstract | 1 |
|  |  | (*b*) Provide in the abstract an informative and balanced summary of what was done and what was found | 3 |
| Introduction | | | |
| Background/rationale | 2 | Explain the scientific background and rationale for the investigation being reported | 4 |
| Objectives | 3 | State specific objectives, including any prespecified hypotheses | 4 |
| Methods | | | |
| Study design | 4 | Present key elements of study design early in the paper | 5 |
| Setting | 5 | Describe the setting, locations, and relevant dates, including periods of recruitment, exposure, follow-up, and data collection | 5 |
| Participants | 6 | (*a*) *Cohort study*—Give the eligibility criteria, and the sources and methods of selection of participants. Describe methods of follow-up  *Case-control study*—Give the eligibility criteria, and the sources and methods of case ascertainment and control selection. Give the rationale for the choice of cases and controls  *Cross-sectional study*—Give the eligibility criteria, and the sources and methods of selection of participants | 5 |
|  |  | (*b*) *Cohort study*—For matched studies, give matching criteria and number of exposed and unexposed  *Case-control study*—For matched studies, give matching criteria and the number of controls per case | 5 |
| Variables | 7 | Clearly define all outcomes, exposures, predictors, potential confounders, and effect modifiers. Give diagnostic criteria, if applicable | 6 |
| Data sources/ measurement | 8* | For each variable of interest, give sources of data and details of methods of assessment (measurement). Describe comparability of assessment methods if there is more than one group | *6* |
| Bias | 9 | Describe any efforts to address potential sources of bias | 6, 11 |
| Study size | 10 | Explain how the study size was arrived at | 7 |
| Quantitative variables | 11 | Explain how quantitative variables were handled in the analyses. If applicable, describe which groupings were chosen and why | 6 |
| Statistical methods | 12 | (*a*) Describe all statistical methods, including those used to control for confounding | 6 |
|  |  | (*b*) Describe any methods used to examine subgroups and interactions | 6 |
|  |  | (*c*) Explain how missing data were addressed | 6 |
|  |  | (*d*) *Cohort study*—If applicable, explain how loss to follow-up was addressed  *Case-control study*—If applicable, explain how matching of cases and controls was addressed  *Cross-sectional study*—If applicable, describe analytical methods taking account of sampling strategy | 6 |
|  |  | (*e*) Describe any sensitivity analyses | 6 |

Continued on next page

| Results | | | |
| --- | --- | --- | --- |
| Participants | 13* | (a) Report numbers of individuals at each stage of study—eg numbers potentially eligible, examined for eligibility, confirmed eligible, included in the study, completing follow-up, and analysed | 8 |
|  |  | (b) Give reasons for non-participation at each stage | 8 |
|  |  | (c) Consider use of a flow diagram | 8 |
| Descriptive data | 14* | (a) Give characteristics of study participants (eg demographic, clinical, social) and information on exposures and potential confounders | 8 |
|  |  | (b) Indicate number of participants with missing data for each variable of interest | 8 |
|  |  | (c) *Cohort study*—Summarise follow-up time (eg, average and total amount) | 8 |
| Outcome data | 15* | *Cohort study*—Report numbers of outcome events or summary measures over time | *8* |
|  |  | *Case-control study—*Report numbers in each exposure category, or summary measures of exposure | *8* |
|  |  | *Cross-sectional study—*Report numbers of outcome events or summary measures | *8* |
| Main results | 16 | (*a*) Give unadjusted estimates and, if applicable, confounder-adjusted estimates and their precision (eg, 95% confidence interval). Make clear which confounders were adjusted for and why they were included | 8 |
|  |  | (*b*) Report category boundaries when continuous variables were categorized | 8 |
|  |  | (*c*) If relevant, consider translating estimates of relative risk into absolute risk for a meaningful time period | 8 |
| Other analyses | 17 | Report other analyses done—eg analyses of subgroups and interactions, and sensitivity analyses | 8 |
| Discussion | | | |
| Key results | 18 | Summarise key results with reference to study objectives | 9,10 |
| Limitations | 19 | Discuss limitations of the study, taking into account sources of potential bias or imprecision. Discuss both direction and magnitude of any potential bias | 11 |
| Interpretation | 20 | Give a cautious overall interpretation of results considering objectives, limitations, multiplicity of analyses, results from similar studies, and other relevant evidence | 9,10 |
| Generalisability | 21 | Discuss the generalisability (external validity) of the study results | 9,10 |
| Other information | | | |
| Funding | 22 | Give the source of funding and the role of the funders for the present study and, if applicable, for the original study on which the present article is based | 2 |

*Give information separately for cases and controls in case-control studies and, if applicable, for exposed and unexposed groups in cohort and cross-sectional studies.

**Note:** An Explanation and Elaboration article discusses each checklist item and gives methodological background and published examples of transparent reporting. The STROBE checklist is best used in conjunction with this article (freely available on the Web sites of PLoS Medicine at http://www.plosmedicine.org/, Annals of Internal Medicine at http://www.annals.org/, and Epidemiology at http://www.epidem.com/). Information on the STROBE Initiative is available at www.strobe-statement.org.

**Details of the structure and functioning of EMS in the present work:**

To access specialized help when a citizen has an emergency (all types of emergencies), they call the emergency telephone number 1-1-2. The appeal is received, geopositioned and referred to the most appropriate manager (medical, police or fire emergency). In the case of a medical emergency, the call is referred as a priority to a specific call-center. A physician and/or emergency nurse coordinator conducts a brief clinical interview to look for signs and symptoms. After this assessment, a case-by-case decision is made on the best strategy to resolve the incident, which may range from telephone medical advice, activation of primary care teams, or if necessary, dispatch of basic life support, advanced life support or helicopter emergency medical service to the scene, or a combination of all of them in particularly complex incidents. The BLS units are staffed by two emergency medical technicians (EMT), while the ALS units comprise two EMTs, an emergency registered nurse (ERN) and a physician.

All the cases analyzed in the present study were patients always evaluated by an ALS and then evacuated to the ED, either in ALS or BLS. On-scene and EMS-provider work with pre-established workflows, following international guidelines appropriate to each pathology. The ALS has the technical capacity to perform advanced airway management, including invasive and non-invasive mechanical ventilation, hemopneumothorax decompression, venous and intraosseous cannulation, bedside or en route drug administration, including fibrinolytic therapy and vasoactive agents, complete hemodynamic monitoring, mobilization and immobilization, use of advanced diagnostic devices, for example, ultrasound or point-of-care testing, etc.

The use of EWS in this context is already a reality. Currently the qSOFA and NEWS2 are used in a standardized manner in patients with suspected infection, the CHA₂DS₂-VASc Score for Atrial Fibrillation Stroke Risk, HAS-BLED Score for Major Bleeding Risk, or the Rapid Arterial oCclusion Evaluation (RACE) Scale for Stroke and the Modified Rankin Scale for Neurologic Disability just to cite a few examples. Once assessed, and with the necessary stabilization at the scene, the patient is referred either by BLS or ALS to the most appropriate center for his or her pathology. Certain time-dependent life-threatening pathologies with specific action codes, i.e., cardiac arrest code, trauma code, stroke code, acute myocardial infarction code, or sepsis code, are preferably evacuated by ALS to the nearest useful center.

**Supplementary material**

Supplementary Table S1. a) Injury severity score, b) Age-adjusted Charlson Comorbidity Index calculation

**a) Injury severity score**

| Standardizes severity of traumatic injury based on worst injury of 6 body systems. | | |
| --- | --- | --- |
| **Head and neck worst injury?** | No injury | 0 |
|  | Minor | 1 |
|  | Moderate | 2 |
|  | Serious | 3 |
|  | Severe | 4 |
|  | Critical | 5 |
|  | Unsurvivable | 6 |
| **Face worst injury?** | No injury | 0 |
|  | Minor | 1 |
|  | Moderate | 2 |
|  | Serious | 3 |
|  | Severe | 4 |
|  | Critical | 5 |
|  | Unsurvivable | 6 |
| **Chest worst injury?** | No injury | 0 |
|  | Minor | 1 |
|  | Moderate | 2 |
|  | Serious | 3 |
|  | Severe | 4 |
|  | Critical | 5 |
|  | Unsurvivable | 6 |
| **Abdomen worst injury?** | No injury | 0 |
|  | Minor | 1 |
|  | Moderate | 2 |
|  | Serious | 3 |
|  | Severe | 4 |
|  | Critical | 5 |
|  | Unsurvivable | 6 |
| **Extremity (including pelvis) worst injury?** | No injury | 0 |
|  | Minor | 1 |
|  | Moderate | 2 |
|  | Serious | 3 |
|  | Severe | 4 |
|  | Critical | 5 |
|  | Unsurvivable | 6 |
| **External worst injury?** | No injury | 0 |
|  | Minor | 1 |
|  | Moderate | 2 |
|  | Serious | 3 |
|  | Severe | 4 |
|  | Critical | 5 |
|  | Unsurvivable | 6 |
| Then, if the 3 most severe injuries in 3 body systems are A, B, and C, then ISS = A² + B² + C²  If a patient has an AIS of 6 in any body system, they are automatically assigned an ISS of 75. | | |

1. Baker SP, O'Neill B, Haddon W Jr, Long WB. The injury severity score: a method for describing patients with multiple injuries and evaluating emergency care. J Trauma. 1974;14(3):187-96.
2. Beverland DE, Rutherford WH. An assessment of the validity of the injury severity score when applied to gunshot wounds. Injury. 1983;15(1):19-22.
3. Copes WS, Champion HR, Sacco WJ, Lawnick MM, Keast SL, Bain LW. The Injury Severity Score revisited. J Trauma. 1988;28(1):69-77.

**b) Age-adjusted Charlson Comorbidity Index calculation**

| **Comorbid condition** | | **Score** |
| --- | --- | --- |
|  | Myocardial infarction | 1 |
|  | Congestive heart failure |  |
|  | Cerebrovascular disease |  |
|  | Peripheral vascular disease |  |
|  | Dementia |  |
|  | Chronic obstructive pulmonary disease |  |
|  | Connective disease |  |
|  | Peptic ulcer disease |  |
|  | Liver disease mild |  |
|  | Diabetes mellitus uncomplicated |  |
|  | Hemiplegia | 2 |
|  | Severe chronic kidney disease |  |
|  | Diabetes mellitus with end organ damage |  |
|  | Solid tumor localized |  |
|  | Leukemia |  |
|  | Lymphoma |  |
|  | Solid tumor metastatic | 3 |
|  | Liver disease severe |  |
|  | Acquired immunodeficiency syndrome | 6 |
| **Age, years** | |  |
|  | 41-50 | 1 |
|  | 51-60 | 2 |
|  | 61-70 | 3 |
|  | ≥ 71 | 4 |

1. Charlson ME, Pompei P, Ales KL, MacKenzie CR. A new method of classifying prognostic comorbidity in longitudinal studies: development and validation. J Chronic Dis. 1987;40(5):373-83.
2. Shuvy M, Zwas DR, Keren A, Gotsman I. The age-adjusted Charlson comorbidity index: A significant predictor of clinical outcome in patients with heart failure. Eur J Intern Med. 2020;73:103-104.

**Data collection and sample size calculations**

Data was collected and registered in a database generated with the IBM SPSS Statistics for Apple version 20.0 software. (IBM Corp, Armonk USA). The caseload entry system was test-run to delete unclear or ambiguous items and to verify the adequacy of the data gathering system. Missing values were completely at random, therefore a listwise deletion method was used since it does not induce to biased means, variances or regression weights modification. The sample size needed for the clustering studies has been recently estimated ​(1)​. Due to the characteristics of the clustering procedure, this is, the phenotypes derived from clustering are driven by large effect sizes or by the accumulation of small effect sizes among the multiple variables analyzed, there is no effect of the covariance structure difference. Therefore, a small samples size (e.g. N=20), as stated in allows large cluster separations ​(1)​.

1. Dalmaijer ES, Nord CL, Astle DE. Statistical power for cluster analysis. BMC Bioinformatics. 2022; 23(1): p. 205.

**Clustering methods**

The clustering was performed by using four different methods based on unsupervised machine learning methods (note that for all the methods the number of clusters was fixed to three based on clinical criteria):

-Clustering method 1 (Alfa): A reduction in dimensionality (principal component analysis) was used to reduce the number of variables. The most parsimonious clustering model was selected by the Bayesian information criterion (BIC) to perform a Gaussian mixture modeling for model-based clustering.

- Clustering method 2 (Beta): The clustering was performed by using K-means clustering, a simple and elegant approach for grouping the data into K-distinct, non-overlapping clusters. The clustering was carried out considering all the available variables and fixing the number of clusters, K, to three based on clinical criteria. After clustering principal component analysis (PCA) was carried out to elucidate the relative importance of each variable in the final cluster assignment.

- Clustering method 3 (Gamma): PCA was first applied to decompose the data in a set of consecutive orthogonal principal components that explain the maximum amount of the variance inherent in the data. The minimum number, V, of principal components required to explain 95% of the variance was identified. Finally, Gaussian Mixture Model (GMM) was used to cluster the first V principal components. GMM assumed that data came from a mixture of Gaussian distributions, which allowed creating clusters with different shapes through a full covariance matrix.

- Clustering method 4 (Delta): Similarly to clustering method 3, PCA was first used to extract the minimum number (V) of principal components that explained 95% of the variance in the data. Those V principal components were then used to group the data into three clusters using K-means.

**Supplementary Results**

Supplemental Figure S1. Chord diagram for mortality and type of trauma for T-1 (a), T-2 (b), and T-3 (c).


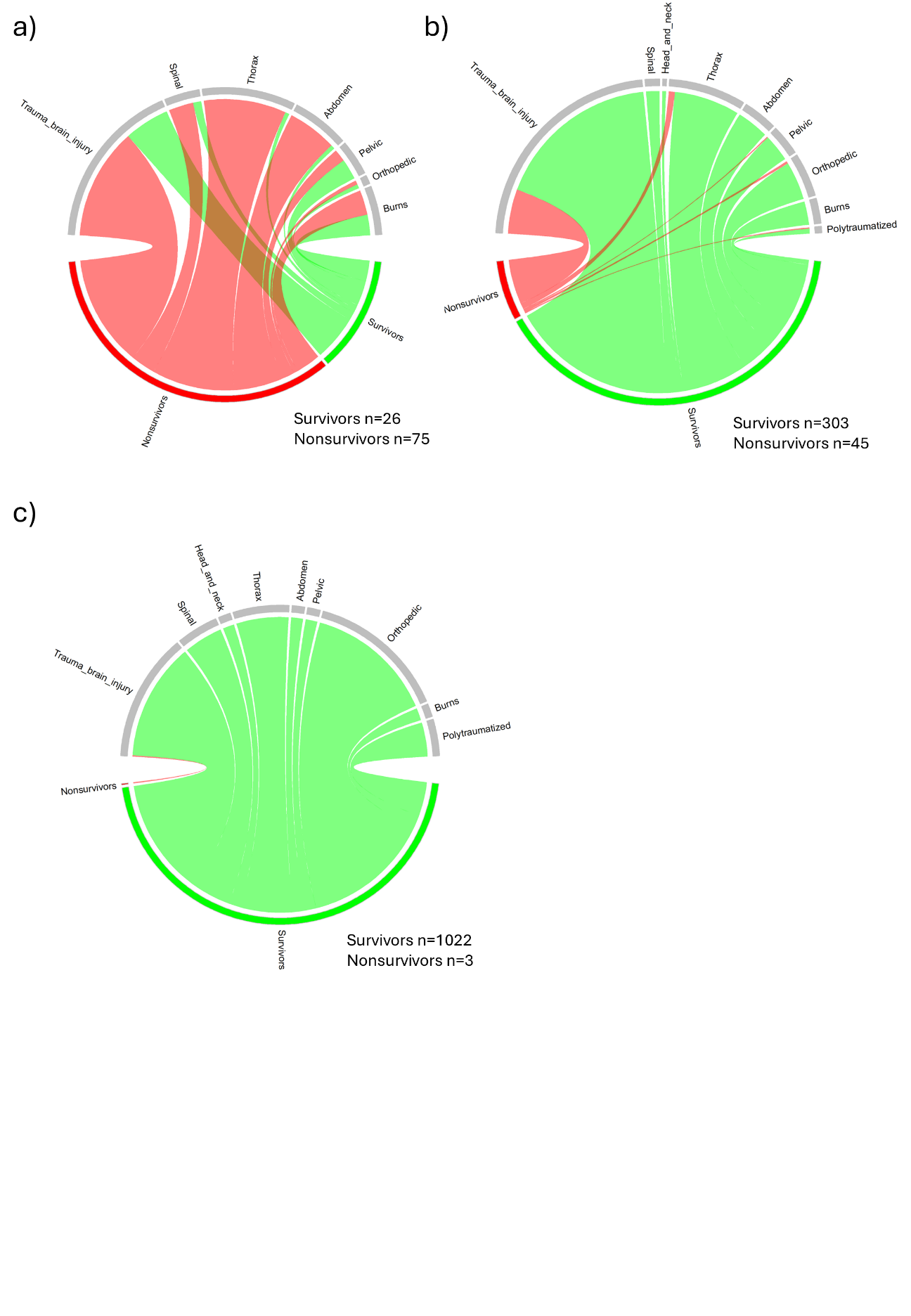


Supplementary Figure S2. A scree plot depicting the percentage of variance explained by each of the principal components.

**
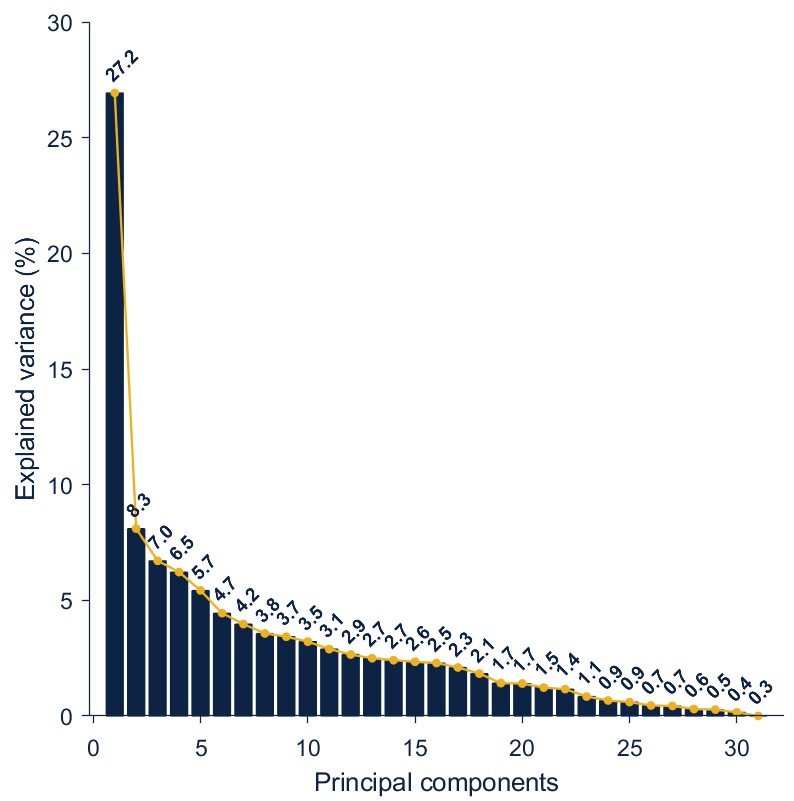
**

Supplementary Figure S3: The first two principal components for the data. The blue/orange/green dots represent the scores for the first two principal components of those patients (observations) assigned to T-1/T-2, and T-3, respectively. The solid red lines indicate the first two principal component loading vectors for each variable.

**
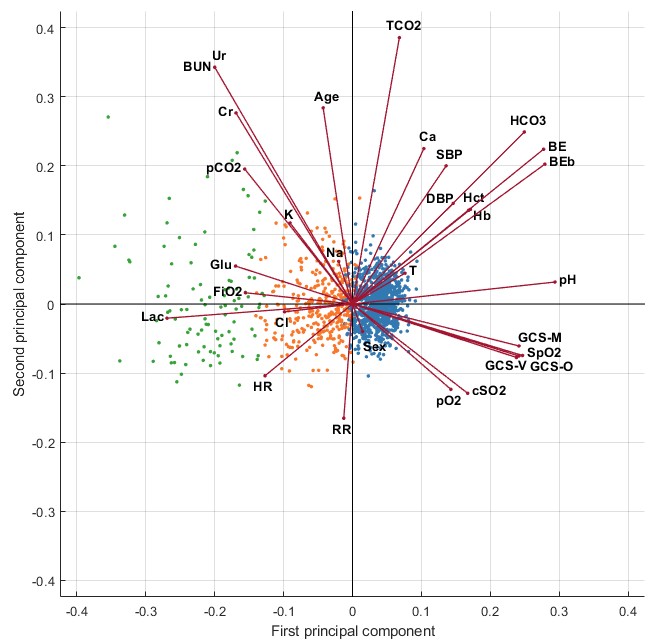
**

Supplementary Figure S4. Heatmap of the loading vectors (φ) of all the principal

components.
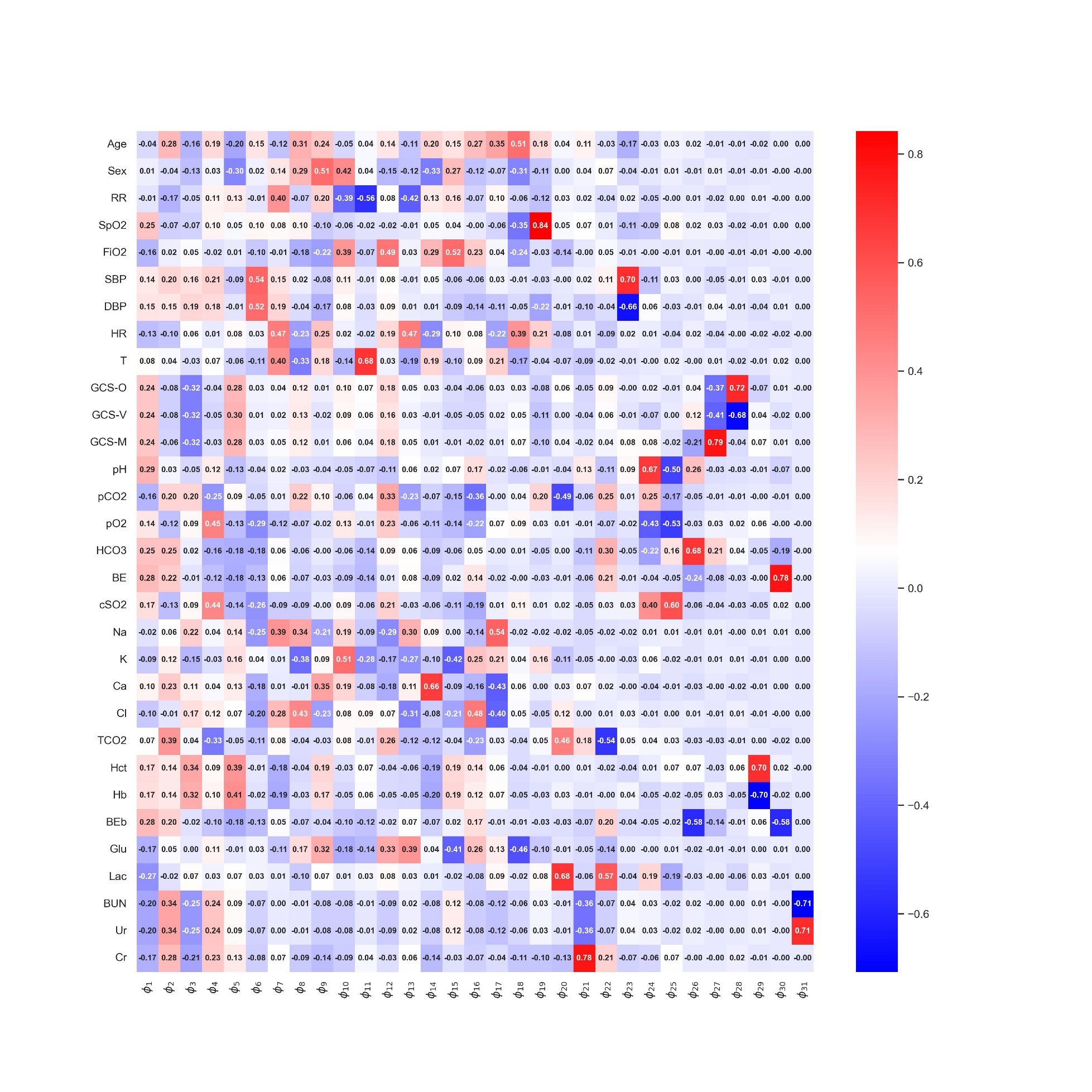

Supplement: Supplementary file 1 — Supplementary Material 1. [file 13049_2026_1553_MOESM1_ESM.docx]
